# Baseline traits of patients presenting at a low vision clinic in Shanghai, China

**DOI:** 10.1186/s12886-015-0013-3

**Published:** 2015-03-03

**Authors:** Guohong Gao, Chaohu Ouyang, Jinhui Dai, Feng Xue, Xiaoying Wang, Leilei Zou, Minjie Chen, Fei Ma, Manrong Yu

**Affiliations:** Department of Ophthalmology, Eye and ENT Hospital Affiliated with Fudan University, Shanghai, China; Key Laboratory of Myopia, Ministry of Health PR China, Shanghai, China; Department of Ophthalmology, Shanghai Peace Eye Hospital, Shanghai, China

**Keywords:** Low vision clinic, Visual impairment, Patient characteristics, Low vision aids, Rehabilitation

## Abstract

**Background:**

Low vision, along with cataract, trachoma, onchocerciasis, childhood blindness and refractive error, is one of the priorities in the global initiative, VISION 2020-The Right to Sight. The purpose of this study was to characterize the traits of patients presenting at a low vision clinic in China.

**Methods:**

A retrospective study was conducted of the records of 299 patients who visited the Low Vision Clinic of Eye and ENT Hospital Affiliated to Fudan University from January 2009 to May 2014. Reviewed parameters included age, gender, education, occupation, cause of visual impairment and types of low vision aids (LVAs) dispensed.

**Results:**

Of all the patients (193 male; aged from 3 to 96 years, with a mean of 29.74 ± 25.23 years), 43.48% experienced moderate visual impairment, 25.42% had severe visual impairment and 21.07% were blind. The four major causes of visual impairment were congenital cataract (14.38%), degenerative myopia (13.71%), juvenile macular degeneration (9.36%) and retinitis pigmentosa (9.36%). The most common causes of visual impairment were congenital cataract (22.67%) in 0-19-year-olds, retinitis pigmentosa (20.62%) in 20-59-year-olds, and age-related macular degeneration (36.54%) in the 60+ group. With the help of LVAs, a significant improvement of distance and/or near vision or visual field was observed in 243 patients, of whom 185 accepted LVAs and 58 patients refused due to high price, inconvenience, young age (≤6 y), clumsy appearance and ignorance. The most commonly dispensed LVAs were stand magnifiers (21.57%) followed by spectacle-type LVAs (19.21%).

**Conclusions:**

The majority of the patients in our low vision clinic were young, the main causes of visual impairment were congenital and hereditary diseases. Stand magnifiers were the most commonly dispensed LVAs. High price was the major reason for refusing LVAs.

## Background

The World Health organization (WHO) reported in 2004 that 314 million people had visual impairment in the world, of whom 45 million were blind, 124 million were classified as having low vision after best correction, and 145 million worldwide were visually impaired due to uncorrected refractive errors [1]. These figures have been reduced with the effective implementation of the program entitled Vision 2020: The Right to Sight and the appropriate interventions. Globally, 285 million people in the world were visually impaired in 2010, of whom 39 million were blind and 246 million had low vision. About 80% of visual impairment can be avoided or cured [2].

Approximately 90% of people with visual impairment live in the developing countries with more than half of them live in Asia and a vast majority in rural communities [2]. Based on surveys conducted in the rural areas combined with data from urban settings, an estimated 75 million Chinese are visually impaired, with 8 million being blind and 67 million having low vision [3-5].

Vision impairment is strongly associated with reduced quality of life [6,7]. It is a common cause of reading difficulty, loss of driving ability, mobility problems, and loss of personal independence [8-11]. It also exerts a significant impact on the socioeconomic development of individuals and societies. Reduced vision brings about significant consequences in relation to the individual, public health, and community, such as increased cost of education, reduced personal income and loss of productivity for those caring for the visually impaired [12,13].

Through the use of low vision aids (LVAs) and rehabilitation therapy, low vision rehabilitation is the primary intervention for visual impairment to improve daily function, seeking to maximize the patient’s ability to perform daily activities, thereby improving quality of life [14-16]. It has been estimated that very few people with visual impairment, possibly only 5-10%, actually use low vision rehabilitation services. Among the established reasons are poor utilization of services, lack of awareness of low vision services among patients, ignorance of rehabilitation services, and low vision referral rates among eye care practitioners [17,18].

The burden of visual impairment is huge; therefore, data on provision of low vision services has become necessary for planning appropriate low vision care and providing quality care to people with visual impairment so that they can restore as much visual function as possible. This paper reports the findings of a retrospective study of low vision clinic population. It is hoped that information obtained would help in policy formulation and funds allocation for low vision services.

## Methods

A retrospective review was conducted of the records of 299 consecutive patients seen at the low vision clinic of Department of Ophthalmology, Eye and ENT Hospital Affiliated to Fudan University between January 2009 and May 2014. Almost all patients were referred from other hospitals. Hospitals of surrounding provinces (e.g. Jiangsu, Zhejiang and Anhui province) preferred to refer people with ocular diseases to a hospital-based low-vision service. The patients had been treated for various diseases for about 6 months but their visual demands were not adequately met by conventional methods. The information extracted from the clinical records of all patients included age, sex, education level, work status, cause of visual impairment, chief visual demand, visual acuity and types of LVAs dispensed.

The study was carried out with approval from the Ethics Committee of the Eye and ENT Hospital of Fudan University in accordance with the tenets of the Helsinki Declaration. All patients gave their informed consent prior to inclusion in this study.

The definitions of visual impairment used for the estimates in this study followed the categories of the International Classification of Diseases Update and Revision 2010 [19].

The causes of visual impairment were determined by ophthalmologists. In cases where two or more disorders might be responsible for the visual impairment, the primary was recorded.

Each patient, based on his or her test results and the daily activity that he or she wanted to achieve in the future, was prescribed more than two LVAs for distance and near vision. LVAs decision was made together by the ophthalmologist, Low-vision therapist, the patient and/or the guardian. Telephone interviews were conducted by the primary author about the ease in using the LVAs 3 months post-intervention.

Data were entered into a database in Microsoft Excel and analyzed using SPSS V.21.0 (Chicago, IL, USA) statistical software.

## Results

Records of 299 patients (193 or 64.55% male) were reviewed. Their ages ranged from 3 to 96 years with a mean of 29.74 ± 25.23. The majority of patients were young with 191 (63.88%) under 30 and 108 (36.12%) over 30. The distribution of age and sex is shown in Table 1.

**Table 1:** **Age and sex distribution**

**Age (years)**	**Sex**		**Total no.(%)**
	**Male**	**Female**	
0-9	56	29	85(28.43)
10-19	42	23	65(21.74)
20-29	25	16	41(13.71)
30-39	12	4	16(5.35)
40-49	14	0	14(4.68)
50-59	13	13	26(8.70)
60-69	6	11	17(5.69)
70-79	16	5	21(7.02)
80+	9	5	14(4.68)
Total	193	106	299(100.00)

The education level, work status and visual demands are summarized in Table 2. The majority of the patients had received school level education at the time of presentation. Most (89.33%) of the young patients (≤19 years) were receiving school education and most (92.31%) of the elderly patients (≥60 years) were retired.

**Table 2:** **Education level, work status and visual demands**

	**Age (years)**									**Total no.(%)**
	**0-9**	**10-19**	**20-29**	**30-39**	**40-49**	**50-59**	**60-69**	**70-79**	**80+**	
**Education Level**										
No education	1	0	0	0	0	0	0	0	0	1(0.33)
<Primary	39	1	0	0	0	0	0	0	0	40(13.38)
Primary	45	29	8	5	4	4	3	1	0	99(33.11)
Middle school	0	23	12	2	4	5	5	2	1	54(18.06)
High school	0	11	2	2	2	8	5	4	5	39(13.04)
College or higher	0	1	19	7	4	9	4	14	8	66(22.07)
**Work Status**										
Students	84	50	9	0	0	0	0	0	0	143(47.83)
Retired	0	0	0	0	0	11	13	21	14	59(19.73)
Job holder	0	5	15	9	10	10	1	0	0	50(16.72)
Unemployed or Dropout	1	10	17	6	1	4	2	0	0	41(13.71)
Farmer	0	0	0	1	3	1	1	0	0	6(2.01)
**Visual Demand**										
Distance vision	19	16	11	1	1	0	0	2	1	51(17.06)
Near vision	21	7	11	5	7	20	15	14	10	110(36.79)
Distance and Near vision	41	42	19	10	5	5	1	5	3	131(43.81)
Visual field expansion	4	0	0	0	1	1	1	0	0	7(2.34)

With regards to the main visual demands, 131(43.81%) patients complained of poor distant and near vision at the same time, indicating that both were of equal importance to them. Among the patients reporting poor distance vision as their main problem, 68.63% were young patients and 5.88% were elderly patients. The patients complained that they could not read books or newspapers, see the contents on a blackboard, watch TV, recognize a person or tell traffic lights at a distance, and other problems because of vision loss.

Of the 299 patients, 30 (10.03%) were classified as with mild visual impairment (≥6/18), 130 (43.48%) moderate (6/18 to 6/60) and 76 (25.42%) severe (6/60 to 3/60), and 63 (21.07%) as blind (<3/60).

The causes of visual impairment are shown in Table 3, with the most common being congenital cataract (22.67%) in 0-19-year-olds, retinitis pigmentosa (20.62%) in 20-59-year-olds, and age-related macular degeneration (AMD, 36.54%) in the 60+ group. Two elderly patients with cataract did not undergo operation because of the poor general health and advanced age.

**Table 3:** **Causes of visual impairment**

**Primary diagnoses**	**Age (years)**									**Total no.(%)**
	**0-9**	**10-19**	**20-29**	**30-39**	**40-49**	**50-59**	**60-69**	**70-79**	**80+**	
Congenital cataract	26	8	6	1	2	0	0	0	0	43(14.38)
Degenerative myopia	5	7	4	2	1	6	7	8	1	41(13.71)
JMD	7	9	9	3	0	0	0	0	0	28(9.36)
Retinitis pigmentosa	1	6	7	3	4	6	0	1	0	28(9.36)
Optic atrophy	7	10	2	3	1	3	1	0	0	27(9.03)
Nystagmus	9	9	3	1	1	0	0	0	0	23(7.69)
Glaucoma	7	4	3	1	0	2	1	3	2	23(7.69)
AMD	0	0	0	0	0	2	3	7	9	21(7.02)
Albinism	8	4	3	0	1	0	0	0	0	16(5.35)
Coloboma	5	2	1	0	0	0	0	0	0	8(2.68)
Diabetic retinopathy	0	0	0	0	1	2	4	0	0	7(2.34)
Trauma	0	1	1	0	2	1	1	0	0	6(2.01)
Retinal detachment	0	1	1	2	0	0	0	1	0	5(1.67)
Corneal opacity	1	1	0	0	0	1	0	1	0	4(1.34)
Cone/Rod dystrophy	1	1	0	0	1	1	0	0	0	4(1.34)
Age-related cataract	0	0	0	0	0	0	0	0	2	2(0.67)
Congenital microphthalmia	2	0	0	0	0	0	0	0	0	2(0.67)
Retinopathy of prematurity	0	1	1	0	0	0	0	0	0	2(0.67)
Optic nerve hypoplasia	2	0	0	0	0	0	0	0	0	2(0.67)
Others*	4	1	0	0	0	2	0	0	0	7(2.34)
Total	85	65	41	16	14	26	17	21	14	299(100.00)

*Abbreviation*: *AMD* age-related macular degeneration, *JMD* juvenile macular degeneration.

*Others: Persistent hyperplastic primary vitreous-1, Familial exudative vitreoretinopathy-1, Morning-glory syndrome-1, Spherophakia-1, Retinoblastoma-1, Hypertensive retinopathy-1, Macular hole-1.

The LVAs (255 for 185 patients) dispensed are listed in Table 4, near LVAs (177, 69.41%) outnumbering distance LVAs (74, 29.02%) and prisms (4, 1.57%).

**Table 4:** **Low vision aids dispensed in relation to age**

**Aid dispensed**	**Age (years)**									**Total no.(%)**
	**0-9**	**10-19**	**20-29**	**30-39**	**40-49**	**50-59**	**60-69**	**70-79**	**80+**	
**Distance**										
Binocular telescope	17	19	3	2	0	0	0	0	0	41(16.08)
Monocular telescope	5	12	9	3	0	2	1	0	1	33(12.94)
**Near**										
Stand magnifier	18	16	6	1	3	2	5	3	1	55(21.57)
Spectacle-type LVA	20	9	3	2	3	5	2	2	3	49(19.21)
Electronic device	12	10	3	6	0	1	2	4	3	41(16.08)
Handheld magnifier	1	4	2	1	1	8	3	4	4	28(10.98)
Binocular telescope	2	2	0	0	0	0	0	0	0	4(1.57)
**Field expansion**										
Prisms	2	1	0	1	0	0	0	0	0	4(1.57)
Total	77	73	26	16	7	18	13	13	12	255(100.00)

With the help of LVAs, a significant improvement of distance and/or near vision or visual field was observed in 243 patients, of whom 185 patients accepted LVAs and 58 refused due to high cost (36.11%), inconvenience (20.83%), young age (≤6 y, 18.06%), clumsy appearance (12.50%), ignorance (6.94%) and discomfort (5.56%). Amongst those who refused LVAs, 17.24% gave two or more reasons. The remaining 56 (18.73%) patients refused to accept LVAs because LVAs did not lead to significant improvement in vision or visual field.

Of all the patients responding to the telephone interviews, 41 (22.16%) reported the use of the aids as easy, 55 (29.73%) relatively easy, 38 (20.54%) a little difficult, 27 (14.59%) very hard, and 24 (12.97%) extremely difficult.

## Discussion

The distribution of age and sex in our study is different from previous reports in the developed countries but similar to those from other developing countries. In this study, a large proportion (73.91%) of patients was below 50 years old, most of the young patients being students who are more likely to seek rehabilitation services because poor vision is a big burden in continuing education. This population composition is similar to the findings from the developing countries [20-25] in which the patients aged below 50 ranged between 57.01% and 73.82% and those aged 60 and above between 18.54% and 31.09%. The high ratio between the male to female in our study is similar to that of other studies conducted in the developing countries (1.83:1 to 2.63:1). In contrast, the majority (>60%) of the patients were aged 60 and above and more females were found to present for low vision services in studies from the developed countries [26-30]. The differences probably suggest the reduced access and utilization of low vision rehabilitation services by females and the older population in the developing countries [24].

Visual impairment could affect work, study and social activities, as mainly evidenced by the premature retirement and unemployment in adults and school dropouts in children. The majority of our patients considered near and distance vision to be of equal importance. The elderly patients were chiefly concerned with reading, while children were more likely to deem distance vision as being their major problem. To meet the visual needs, it is very important to consider a patient’s working distance and the desired outcome when prescribing LVAs.

The most common causes of visual impairment in our study population as a whole differed markedly from the studies in Korea (optic atrophy) [20], India (retinitis pigmentosa) [21] and Malaysia (structural defects) [22]. Two studies in Nepal [23,25] reported the most common causes in the country were diabetic retinopathy and retinitis pigmentosa. In contrast, studies in the developed countries [26-30] found AMD as the most common cause. The possible reasons for lower prevalence of AMD in the developing countries may include age and nutrition, less cigarette smoking, and lower body mass index [24,31]. In our study, the most common causes of visual impairment among children were congenital and hereditary diseases, which is similar to other reports [22-24,27]. Retinitis pigmentos was found to be a the leading cause among the adults aged 20–59 years. Both in our study and the studies in Malaysia [22] and Nigeria [24]. AMD was identified as the most common cause of visual impairment among those over 60 in our study and in other studies [20,22-24,26-30]. Different from other studies, degenerative myopia was the the second leading cause of visual impairment in our study and also the first cause of visual impairment in patients aged 20 and above. Considering the high prevalence of myopia in China, the visual impairment caused by myopia deserves more attention. In our study, the majority of the patients with congenital cataract, who unresponsive to therapeutic training for amblyopia, were companied by nystagmus, strabismus and secondary glaucoma. These findings suggest that great emphasis should be placed on the rehabilitation in the patients with congenital cataract combined with other eye diseases.

Spectacle-type LVAs came second in our study in contrast to the findings of India [21] and Nepal [23] in which spectacle magnifiers were the most commonly dispensed devices. Handheld magnifiers were dispensed less frequently in our clinic than in others [20,22,23,27-30]. Stand magnifiers and spectacle-type LVAs were the most commonly used LVAs for near vision, while binocular telescopes were the most popular devices for distance vision. Dispensing LVAs reflected the visual demands of patients, with the majority being dispensed to those under 60 years of age. In most LVA holders, visual acuity (near and/or distance) or visual field were much improved when using the LVAs, suggesting that LVAs were very helpful devices to patients with visual impairment. However, a considerable number of patients would give up the long-term use of the LVAs. The results of the telephone survey showed that about half of LVAs holders could not utilize LVAs effectively, which might be related to disease progression, physical deterioration, poor compliance and mobility of patients, and inadequate guidance and training in the use of LVAs.

In this study, we found that availability, portability, durability and appearance of the LVAs, and affordability, cognitive ability and age of the patients were the main influencing factors in dispensing LVAs. Generally, electronic magnifiers could improve visual function as good as, even better than optical magnifiers. However, electronic devices were dispensed less frequently mainly because they were too expensive. Besides, the standby time was short, and it was difficult to repair if they were damaged. Some patients refused LVAs because their vision was either too poor or too good. The majority of the LVAs refused by patients because of high price were electronic devices and binocular telescopes. Inconvenience of LVAs was mainly reflected in poor portability and operation difficulty. Most of the patients who refused to accept LVAs for being too young were preschoolers, and they shared a number of common characteristics such as poor independence, noncompliance and less visual demand for reading and seeing a blackboard. Some parents preferred environmental modifications such as moving the children closer to the chalkboard or asking them to hold the book closer. For the cosmetic reasons, spectacle-type devices and binocular telescope were primarily refused by patients. The patients who accepted LVAs shared some of the features such as definite visual demands, good affordability, good cognitive ability and significant improvement in the quality of life with the help of LVAs.

The present retrospective study has a number of limitations that should be acknowledged. First, it had a low response rate and lacked long-term follow-up data with fewer than a third of the patients responding to the interview after rehabilitation. Second, we used a convenience sample, and as such they may not be representative of all patients with visual impairment in China. Third, the small sample size limited the significance of the results. Despite these limitations, this study is among the first to provide hospital-based data on visual impairment in China. Such data, particularly the findings that the subjects with visual impairment refused useful LVAs in this setting, are of value in planning strategies to improve the eye health in China, who has the largest population in the world.

## Conclusions

In summary, patients seeking low vision rehabilitation services are predominantly young and male in our low vision clinic. Congenital and hereditary diseases were the main causes of visual impairment, while age related diseases (e.g., AMD) were the most common causes among the elderly. Screening in the communities and schools is useful for early detection, diagnosis and treatment of these diseases. The growing focus on visual impairment, rehabilitation and education means that eye care/healthcare providers need to take the lead in working with the education and rehabilitation communities. Counselling, a critical component of eye care, should be provided throughout the program. In addition, great emphasis should be focused on popularization, education and guidance of low vision rehabilitation, which will heighten awareness of the accessibility and availability of low vision care.

As we have discussed, LVAs can be of great help in the daily lives, work and study of patients with visual impairment; however, consistent effort and cooperation between patients and doctors are required. The rehabilitation of low vision is challenging, requiring a multidisciplinary team of ophthalmologists, optometrist, consultants and rehabilitation therapists. According to other studies [16,32-34], patients with visual impairment should be referred to multidisciplinary specialist centers and encouraged to accomplish regular follow-up and training. The key to the success of the LVAs is to keep them portable, comfortable, aesthetic and inexpensive, according to the study. It is, we propose, most important to revamp of the medical insurance coverage in Low vision rehabilitation.
